# Gender-specific differences in the incidence of microalbuminuria in metabolic syndrome patients after treatment with fimasartan: The K-MetS study

**DOI:** 10.1371/journal.pone.0189342

**Published:** 2017-12-19

**Authors:** Jeong Bae Park, Su-A Kim, Ki-Chul Sung, Jang Young Kim

**Affiliations:** 1 JB lab and clinic, Seoul, South Korea; 2 Department of Medicine, Cheil General Hospital, Dankook University College of Medicine, Seoul, South Korea; 3 Department of Critical Care Medicine, Samsung Medical Center, Sungkyunkwan University School of Medicine, Seoul, South Korea; 4 Division of Cardiology, Department of Internal Medicine, Kangbuk Samsung Hospital, Sungkyunkwan University School of Medicine, Seoul, South Korea; 5 Division of Cardiology, Department of Internal Medicine, Yonsei University Wonju College of Medicine, Gangwon-do, Republic of Korea; The University of Tokyo, JAPAN

## Abstract

**Background:**

The effect of resolving metabolic syndrome on target organ damage in hypertensive patients is not well described. We evaluated whether treating metabolic syndrome (MetS) with an angiotensin receptor blocker subsequently reduced microalbuminuria in the K-MetS cohort.

**Methods:**

Among 10,601 total metabolic syndrome patients, 3,250 (52.2% male, 56.2±10.0 years) with sufficient data on five specific metabolic components were included in this study. Patients were divided into four groups based on MetS status at baseline and 3 months. All patients received an angiotensin receptor blocker, fimasartan, for these 3 months; thereafter, treatment was modified at the discretion of each patient’s physician. Microalbuminuria and the albumin/creatine ratio were evaluated as a proxy of organ damage.

**Results:**

Blood pressure and waist circumference decreased from baseline to 3 months and 1 year. The average albumin/creatinine ratio significantly improved during the first three months of the study from 36.0±147.4 to 21.0±74.9 mg/g (p<0.05) and was persistently high in patients with MetS at baseline and 3 months versus other groups. Women in comparison with men showed significantly lower ACR among patients with newly developed MetS at 3-month.

**Conclusions:**

Treatment of hypertensive patients for one year with the angiotensin receptor blocker fimasartan significantly reduced the albumin/creatine ratio, irrespective of whether the patient had MetS; however, the albumin/creatinine ratio was significantly higher in patents with persistent or newly developed MetS compared to patients without MetS. Additionally, these findings were more prominent in women than in men.

## Introduction

Metabolic syndrome (MetS) is a group of metabolic risk factors that result in prothrombotic and proinflammatory conditions[1] that promote the development of cardiovascular disease (CVD)[2, 3]. These metabolic risk factors include dyslipidemia, hypertension, and insulin resistance. Central obesity, hypertension, high fasting blood glucose levels, high serum triglycerides, and low HDL[4, 5] function have been established by NCEP’s ATP III guidelines as risk factors for the development of various atherosclerotic vascular diseases (ASCVD), each with varying degrees of association.[3, 6, 7, 8] Although MetS is particularly prevalent in hypertensive patients[9], MetS is becoming especially more prevalent in Asian populations due to an increased incidence of high-fat diet and sedentary behaviors among this group[10, 11].

Previous studies have shown that hypertension is the most prevalant metabolic risk factor in individuals with MetS[12, 13]. Furthermore, the risk for adverse cardiovascular events is higher in hypertensive individuals than in normotensive individuals, and this risk increases as the number of MetS risk factors increases[14]. Therefore, lowering high blood pressure in patients with MetS may be an effective means to not only relieve MetS but also reduce the associated cardiovascular risks. Various antihypertensive agents are available, but there is no “gold standard” treatment for hypertensive patients with MetS. Beta blockers and diuretics have been associated with less favorable metabolic profiles and increased incidence of diabetes[15–17]. Conversely, calcium channel blockers (CCBs), angiotensin receptor blockers (ARBs), and angiotensin-converting enzyme (ACE) inhibitors are metabolically neutral. Addtionally, several studies have reported improvement in glucose metabolism, inflammation, endothelial dysfunction, and delay of type 2 diabetes with ARB treatment[18–20]. Nevertheless, there are no studies describing the therapeutic effects of ARBs on MetS, associated organ damage, or the subsequent development of CVD. Additionally, there is no clear evidence suggesting that the correction of metabolic syndrome in hypertensive patients reduces organ damage or CVD incidence. Therefore, we evaluated whether controlling MetS in hypertensive patients through the ARB fimasartan reduced the albumin/creatinine ratio (ACR; a proxy for organ damage) and microalbuminuria, a strong predictor of future cardiovascular events.

## Materials and methods

### Study participants

The present research is part of the K-MetS study, a prospective, multicenter, two-arm, observational study examining the effects of early reduction of blood pressure and the correction of MetS on the development of major adverse cardiovascular diseases (MACE) and organ damage in hypertensive patients using the angiotensin receptor blocker fimasartan. The study design has been described in detail elsewhere[21]. Briefly, study participants were enrolled from 593 primary clinics and tertiary University hospitals nationwide in South Korea. Subjects were hypertensive patients prescribed fimasartan, and the dosage and frequency were at the discretion of the treating physician. The total follow-up time was 1 year. The study protocol was approved by the Institutional Review Board (CGH-IRB-2011-54). Committee at the Cheil General Hospital, Dankook University College of Medicine, on behalf of primary and secondary clinics, and institutional review board committees at 10 other University hospitals. All patients gave written informed consent.

### Study design

The K-MetS Cohort Study recruited a total of 10,601 patients from 582 primary clinics and secondary hospitals and 11 University hospitals from October 17, 2011, to October 31, 2012. Patients diagnosed with hypertension and at least 20 years of age were enrolled in the study. Participants that made follow-up visits at 3 month and 1 year (which required a fasting period prior to the visit), during which the ACR and risk factors comprising MetS were measured, remained enrolled in the study. Patients treated with fimasatan at the baseline were excluded. A total of 3,250 patients with completed measurements on MetS risk factors and the ACR at baseline, 3 months, and 1 year were included in the analysis. The patients were divided into four groups by MetS status at baseline and after three month of fimasartan treatment: MetS at both baseline and 3 months, MetS (+/+); MetS at baseline but not at 3 months, MetS (+/-); no MetS at baseline and MetS at 3 months, MetS (-/+); and no MetS at either timepoint, MetS (-/-).

### Definition of metabolic syndrome

The definition of MetS in this study followed the harmonized definition previously reported[22]: 1) systolic blood pressure (SBP) ≥130 mmHg and/or diastolic blood pressure ≥85 mmHg, or antihypertensive drug treatment, 2) fasting blood glucose level ≥100 mg/mL or drug treatment for elevated glucose, 3) waist circumference ≥ 90 cm for men or ≥ 80 cm for women, following Asian-specific cutoffs[5], 4) high-density lipoprotein cholesterol (HDL-C) <40 mg/dL for men and < 50 mg/dL for women, or drug treatment for reduced HDL-C, and 5) triglycerides ≥ 150 mg/dL or drug treatment for elevated triglycerides. If at least three of the above five criteria were met, a subject was diagnosed with metabolic syndrome[5].

### Blood pressure measurements

Blood pressure was measured at the clinics under standardized conditions, with the same nurse or physician using the same instrument to take the measurement on a participant at every time point. All clinics used the Omron HEM-7220 (Omron, Tokyo, Japan), an upper-arm cuff device based on the cuff-oscillometric principle. Blood pressure was measured at least twice on the same arm at each visit after a 5-minute seated rest. The average value of the two measurements was used for analysis.

### Laboratory analysis of blood and urine samples

All clinics and hospitals used a central laboratory (Green Cross Reference Lab, Korea) for analyses of blood and urine samples. Blood was collected from patients after fasting for at least eight hours, and complete blood counts were performed using an automated hematology analyzer (Sysmex SE9000, Toa Medical Electronics, Kobe, Japan). Serum sodium and potassium concentrations were determined by an indirect ion-specific electrode on a Roche Modular ISE 900 (Roche Diagnostics, Mannheim, Germany). Plasma levels of total cholesterol, triglycerides, HDL-C, and low-density lipoprotein cholesterol (LDL-C) were analyzed with the Dimension Clinical Chemistry System (Dade Behring Inc. Newark, DE 19714, U.S.A), Roche Elecsys 2010, and Modular Analytics E170 (Elecsys module) immunoassay analyzers (Roche Diagnostics GmbH, D-68298 Mannheim), respectively. Urinary albumin and creatinine were measured from fasting spot urine samples by immunoturbidimetric assay, using a Roche Cobas Integra 800 chemistry analyzer (Roche Diagnostic, GmbH, Mannheim, Germany). All measurements were done on samples collected at baseline, 3 months, and 1 year. We also evaluated the dependence of urinary ACR and microalbuminuria on metabolic syndrome.

### Statistical analyses

General statistics of the study population are presented as the mean ± standard deviation (SD) for continuous variables, and as frequencies and percentages (%) for categorical variables. The ACR was log transformed to satisfy the normal distribution. Continuous variables were compared using an independent t-test or analysis of variances (ANOVA) with Bonferroni correction for multiple comparison. When significant effects were found with ANOVA, we performed a multiple comparison test with Tukey’s test. Categorical variables were compared using the chi-squared test. Change in the measured variables(basline, 3-month, 1-year) were evaluated using one-way repeated measure ANOVA and post-hoc analysis using Tukey’s test. A two-way repeated measure ANOVA was performed to compare the effect of time and that of group(four Mets group) on ACR. The adjusted least square (LS) means of the ACR at 1 year and ACR variation (baseline-1-year) were compared between groups after adjusting for age, sex, body mass index, diabetes mellitus, baseline ACR, and SBP in the analysis of covariance (ANCOVA) model. Then, multiple logistic regression analysis was performed to assess the effect of MetS status at 3 months on microalbuminuria 1 year later after adjusting for the above-mentioned covariates. A p-value of less than 0.05 was considered significant. All statistical analyses were conducted using SAS software (version 9.3, SAS Institute Inc., Cary, NC, USA).

## Results

### Baseline characteristics

A total of 3,250 patients were enrolled in the study. The mean age of the study population was 56.2 ± 10.0 years and 52.2% of patients were men. A total of 1,843 patients (57%; 56.5% of men and 57% of women) were diagnosed with metabolic syndrome. Both men and women with MetS had a higher body mass index (BMI), larger waist circumference, fasting glucose, triglyceride, lower HDL-cholesterol, and percentage of microalbuminuira than patients without MetS. Patients with MetS were also more likely to have diabetes than those without MetS. Further, MetS patients were more likely to be smokers in men, and had a longer history of hypertension, and a higher elevation of ACR in women (Table 1). Frequency of antihypertensive and other MetS-affecting drugs taken at baseline, 3-month and 1-year is shown in S1 Table.

**Table 1:** Gender-stratified patient baseline characteristics.

	Patients (N = 3,250)	Men (N = 1,697)			Women (N = 1,553)		
		MetS (+) (N = 958)	MetS (-) (N = 739)	p-value	MetS (+) (N = 885)	MetS (-) (N = 668)	p-value
Age, year	56.2 ± 10.0	54.9 ± 10.2	55.2 ± 10.5	1.0000	58.3 ± 9.7	56.4 ± 8.9	0.0003
Height, cm	162.6 ± 8.6	169.1 ± 5.9	168.4 ± 5.7	0.0288	155.8 ± 5.5	155.7 ± 5.4	1.0000
Weight, Kg	67.5 ± 11.3	75.8 ± 10.3	69.2 ± 8.8	<.0001	63.8 ± 9.5	58.7 ± 8.3	<.0001
Body mass index, Kg/m^2^	25.5 ± 3.2	26.5 ± 2.9	24.3 ± 2.5	<.0001	26.3 ± 3.6	24.2 ± 3.0	<.0001
Waist,cm	87.2 ± 8.9	92.7 ± 7.7	85.6 ± 6.6	<.0001	87.5 ± 8.4	80.7 ± 8.4	<.0001
Systolic blood pressure, mmHg	143.7 ± 17.2	145.0 ± 16.7	143.2 ± 16.25	0.0927	143.3 ± 17.6	143.1 ± 18.3	1.0000
Diastolic blood pressure, mmHg	88.4 ± 11.4	89.6 ±11.7	88.90 ± 11.21	0.7230	87.2 ± 11.0	88.0 ± 11.4	0.4224
Fasting glucose, mg/dL	106.3 ± 34.7	121 ± 45.6	97.6 ± 24.4	<.0001	109.5 ± 31.9	90.7 ± 14.4	<.0001
Triglycerides, mg/dL	157.4 ± 98.6	215.6 ± 115.8	118.8 ± 66.0	<.0001	170.1 ± 93.6	100.2 ± 40.8	<.0001
HDL cholesterol, mg/dL	52.0 ± 14.0	44.2 ± 11.7	54.7 ± 12.0	<.0001	49.3 ± 12.5	63.6 ± 12.5	<.0001
Current smoking, %	590 (18.2)	330 (34.5)	217 (29.4)	0.0189	28 (3.2)	15 (2.3)	0.1653
FHx of CVD*, %	595 (18.3)	155 (16.2)	134 (18.1)	0.9894	159 (18.0)	147 (22.0)	0.3822
Hypertensive history, years	3.9 ± 5.2	4.2 ± 5.2	3.8 ± 5.3	0.3333	4.2 ± 5.4	3.4 ± 4.6	0.0063
Estimated GFR, ml/min	102.9 ± 16.9	102.4 ± 18.2	102.9 ± 16.9	1.0000	92.0 ± 16.6	92.5 ± 15.3	1.0000
ACR, mg/g	36.0 ± 147.4	44.7 ± 208.8	29.0 ± 109.7	0.1380	42.3 ± 134.1	22.9 ± 76.2	0.0009
Log(ACR)	2.4 ± 1.2	2.4 ± 1.3	2.1 ± 1.2	0.1910	2.6 ± 1.3	2.3 ± 1.0	0.0010
Microalbuminuria, n (%) (ACR≥30 mg/g)	555 (17.1)	174 (18.2)	102 (13.8)	0.0474	185 (20.9)	94 (14.1)	0.0015
**Concomitant Disease**							
Diabetes, n (%)	579 (17.8)	271 (28.3)	79 (10.7)	<.0001	210 (23.7)	19 (2.8)	<.0001
Ischemic heart disease, n (%)	158 (4.9)	47 (4.9)	42 (5.7)	1.0000	44 (5.0)	25 (3.7)	0.7332
Stroke, n (%)	24 (0.7)	11 (1.2)	2 (0.3)	0.1194	8 (0.9)	3 (0.5)	0.8700
Add-on, n (%)	864 (26.6)	293 (30.6)	200 (27.1)		224 (25.3)	147 (22.0)	

mean ± SD, frequency (%); p-value, Independent t-test with Bonferroni correction;

Mets (+), Mets at baseline; Mets (-), no Mets at baseline.

* Family history of cardiovascular disease, ACR, albumin:creatinine ratio

### Fimasartan-associated changes in BP, metabolic syndrome, and ACR

Several risk factors improved in both male and female patients after three months of treatment with fimasartan. In men, systolic blood pressure decreased significantly from 144.5 ± 16.5 mmHg at baseline to 127.8 ± 12.6 mmHg at 3 months, and remained steady at 127.8 ± 12.3 mmHg at 1 year (p < 0.001, repeated measure ANOVA). A similar pattern was observed in women (143.5 ± 17.8 mmHg at baseline, 126.0 ± 13.5 mmHg at 3 months, and 125.6 ± 12.7 mmHg at 1 year (p < 0.0001, repeated measure ANOVA)). Waist circumference also significantly decreased at 3 months and remained significantly reduced for the entire study duration. Triglycerides also significantly reduced, but only at 1 year. Improvement of fasting glucose and HDL cholesterol was noted at 3 months, but was not maintained. Additionally, after 3 months of treatment with fimasartan, the ACR significantly decreased from 37.9 ± 172.9 mg/g and 33.9 ± 113.2 mg/g to 23.1 ± 92.4 mg/g and 18.8 ± 49.1 mg/g in men and women, respectively, and remained steady until 1 year (Table 2).

**Table 2:** Gender-stratified changes in metabolic components after fimasartan treatment.

	Patients (N = 3,250)				Men (N = 1,697)				Women (N = 1,553)				Men vs. Women p-value^b^
	Baseline	3-month	1-year	P-value^a^	Baseline	3-month	1-year	P-value^a^	Baseline	3-month	1-year	P-value^a^	
SBP, *mmHg*	143.7 ± 17.2	126.9 ± 13.1*	126.7 ± 12.6*	<.0001	144.5 ± 16.5	127.8 ± 12.6*	127.8 ± 12.3*	<.0001	143.5 ± 17.8	126.0 ± 13.5*	125.6 ± 12.7*	<.0001	0.2120
DBP, *mmHg*	88.4 ± 11.4	79.4 ± 9.3*	78.6 ± 8.8*†	<.0001	89.5 ± 11.5	79.9 ± 9.4*	79.3 ± 9.0*†	<.0001	87.8 ± 11.2	78.8 ± 9.0*	77.9 ± 8.5*†	<.0001	0.4679
Waist circumference, cm	87.2 ± 8.9	86.8 ± 8.8*	86.8 ± 9.1*	<.0001	89.6 ± 8.0	89.3 ± 7.98*	89.1 ± 8.2*	<.0001	84.6 ± 9.1	84.1 ± 9.0*	84.3 ± 9.4*	<.0001	0.0446
FBS, mg/dL	106.3 ± 34.7	104.3 ± 29.8*	107.0 ± 32.0†	<.0001	110.8 ± 39.7	107.3 ± 32.0*	110.1 ± 34.6†	<.0001	101.4 ± 27.5	101.1 ± 26.8	103.6 ± 28.6*†	0.0009	0.0038
Triglyceride, mg/dL	157.4 ± 98.6	157.1 ± 92.3	145.7 ± 77.3*†	<.0001	173.4 ± 108.5	172.2 ± 102.2	155.6 ± 82.2*†	<.0001	140 ± 83.1	140.8 ± 76.7	134.8 ± 69.9*†	0.0092	<.0001
HDL, mg/dL	52.0 ± 14.0	51.9 ± 13.8	52.6 ± 13.9*†	0.0003	48.8 ± 12.9	49.0 ± 12.8	49.8 ± 13.0*†	0.0003	55.4 ± 14.4	55.0 ± 14.1	55.6 ± 14.2†	0.0657	0.0623
Metabolic syndrome, n (%)	1843 (56.7)	1824 (56.1)	1828 (56.3)	0.7728	958 (56.5)	932 (54.9)	932 (54.9)	0.3479	885 (57.0)	892 (57.4)	896 (57.7)	0.8536	0.3710
Estimated GFR, ml/min	97.7 ± 17.7	83.9 ± 15.8*	94.9 ± 19.4*†	<.0001	102.6 ± 17.7	88.8 ± 16.2*	100.2 ± 19.8*†	<.0001	92.3 ± 16.1	78.6 ± 13.4*	89.1 ± 17.1*†	<.0001	0.2303
ACR, *mg/g*	36.0 ± 147.4	21.0 ± 74.9*	23.3 ± 95.4*	<.0001	37.9 ± 172.9	23.1 ± 92.4*	24.7 ± 89.5*	<.0001	33.9 ± 113.2	18.8 ± 49.1*	21.8 ± 101.4*	<.0001	0.9041
Log (ACR)	2.4 ± 1.2	2.1 ± 1.1*	2.1 ± 1.2*	<.0001	2.2 ± 1.3	2.0 ± 1.2*	2.0 ± 1.2*	<.0001	2.5 ± 1.2	2.2 ± 1.0*	2.2 ± 1.0*	<.0001	0.0784
Microalbuminuria, n (%) (ACR ≥ 30 mg/g)	555 (17.1)	343 (10.6)*	366 (11.3)*	<.0001	276 (16.3)	185 (10.9)*	206 (12.1)*	<.0001	279 (18.0)	158 (10.2)*	160 (10.3)*	<.0001	0.3113

p-value^a^; changes in metabolic components were assessed with repeated measures ANOVA.

p-value^b^; comparison of changes in metabolic components between men and women using repeated meaures ANOVA.

Comparisons significant at the α = 0.05 level are indicated by *, difference with respect to baseline; †, with respect to 3-month follow-up; *†, with respect to baseline and 3-month follow-up(post-hoc analysis using Tukey’s test).

SBP, systolic blood pressure; DBP, diastolic blood pressure; FBS, fasting blood sugar; HDLc, high-density lipoprotein cholesterol

Different changes in individual MetS risk factors were observed in each of the four study groups. All risk factors except triglycerides slightly improved at 3 months in MetS (+/+) patients, and all components improved at year 1 in this group. Opposite trends were observed in the MetS (+/-) and MetS (-/+) groups; all metabolic components improved at 3 months in the MetS (+/-) group with maintained improvement at 1 year while all metabolic components showed deterioration in the MetS (-/+) group at 3 months. Similarly, in the MetS (-/-) group, there was no improvement in fasting glucose, triglycerides, or HDL cholesterol after 3 months, with a general worsening of all MetS components after 1 year (Table 3).

**Table 3:** Changes in metabolic components after fimasartan treatment across the four study groups.

	MetS (+/+) (N = 1,460)				MetS (+/-) (N = 383)				MetS (-/+) (N = 364)				MetS (-/-) (N = 1,043)			
	Baseline	3-month	1-year	P-value	Baseline	3-month	1-year	P-value	Baseline	3-month	1-year	P-value	Baseline	3-month	1-year	P-value
SBP, *mmHg*	144.6 ± 17.0	127.5 ± 13.1*	127.3 ± 12.5*	<.0001	143.9 ± 17.5	126.9 ± 12.3*	127.2 ± 12.2*	<.0001	143.1 ± 16.7	125.8 ± 13.2*	125.6 ± 12.0*	<.0001	143.5 ± 17.4	126.6 ± 13.3*	126.1 ± 12.9*	<.0001
DBP, *mmHg*	88.7 ± 11.4	79.6 ± 9.4*	78.8 ± 8.4*†	<.0001	88.3 ± 11.8	79.4 ± 8.9*	78.5 ± 8.4*	<.0001	89.0 ± 11.3	79.0 ± 9.2*	78.4 ± 8.7*	<.0001	88.6 ± 11.3	79.2 ± 9.1*	78.5 ± 9.3*	<.0001
Wt,kg	70.8 ± 11.8	70.6 ± 12.1*	70.6 ± 11.9*	<.0001	67.2 ± 10.1	66.7 ± 10.1*	66.9 ± 10.1	0.0004	66.4 ± 10.7	66.4 ± 10.7	66.5 ± 10.5	0.6633	63.4 ± 9.7	63.1 ± 9.6*	63.4 ± 9.7†	<.0001
Waist, cm	90.9 ± 8.4	90.4 ± 8.4*	90.2 ± 8.7*	<.0001	87.8 ± 8.0	86.5 ± 7.9*	87.1 ± 8.4*†	<.0001	85.9 ± 7.9	86.4 ± 7.9*	85.8 ± 8.1†	0.0088	82.4 ± 7.7	82.0 ± 7.6*	82.3 ± 8.2	0.0039
FBS, mg/dL	117.8 ± 42.9	114.6 ± 35.5*	115.8 ± 38.7	0.0046	106.5 ± 24.8	95.7 ± 19.2*	103.4 ± 26.5*†	<.0001	95.8 ± 21.8	104.4 ± 25.4*	104.3 ± 25.4*	<.0001	93.8 ± 20.1	93.1 ± 18.6	96.9 ± 20.1*†	<.0001
Triglyceride, mg/dL	201.4 ± 115.0	196.9 ± 103.2	170.3 ± 4.7*†	<.0001	164.7 ± 69.6	119.9 ± 48.7*	142.3 ± 67.1*†	<.0001	121.7 ± 65.9	178.8 ± 84.2*	147.4 ± 75.7*†	<.0001	105.9 ± 51.8	107.7 ± 54.5	112.0 ± 54.3*†	0.0018
HDL, mg/dL	45.5 ± 11.9	46.1 ± 12.4*	47.8 ± 12.7*†	<.0001	51.1 ± 12.9	55.1 ± 11.5*	53.9 ± 13.2*	<.0001	55.8 ± 12.5	49.5 ± 12.7*	51.9 ± 12.7*†	<.0001	60.1 ± 13.1	59.6 ± 12.7	59.0 ± 13.4*†	0.0059
Estimated GFR, ml/min	97.3 ± 18.4	83.3 ± 16.3*	94.6 ± 20.0*†	<.0001	97.8 ± 17.4	83.9 ± 14.8*	94.2 ± 17.5*†	<.0001	98.1 ± 17.9	84.7 ± 14.9*	95.1 ± 19.6*†	<.0001	97.9 ± 16.6	84.4 ± 15.7*	95.4 ± 19.0*†	<.0001
ACR, mg/g	47.9 ± 192.7	26.8 ± 96.3*	30.1 ± 123.8*	<.0001	26.8 ± 93.0	18.3 ± 51.0	19.5 ± 49.3	0.0518	31.0 ± 119.0	17.1 ± 47.8*	20.0 ± 62.0	0.0187	24.4 ± 85.5	15.2 ± 51.7*	16.4 ± 67.6*	<.0001
Log (ACR)	2.5 ± 1.3	2.2 ± 1.2*	2.2 ± 1.2*	<.0001	2.3 ± 1.1	2.0 ± 1.1*	2.1 ± 1.1*	<.0001	2.2 ± 1.2	2.0 ± 1.0*	2.0 ± 1.1*	<.0001	2.2 ± 1.1	1.9 ± 1.0*	1.9 ± 1.0*	<.0001
Microalbuminuria, %	296 (20.3)	190 (13.0)*	204 (14.0)*	<.0001	63 (16.5)	41 (10.7)*	44 (11.5)*	0.0026	51 (14.0)	39 (10.7)	41 (11.3)	0.1352	145 (13.9)	73 (7.0)*	77 (7.4)*	<.0001

mean ± SD, frequency (%); p-value; changes in metabolic components were assessed repeated measures ANOVA.

Comparisons significant at the α = 0.05 level are indicated by *, difference with respect to baseline; †, with respect to 3-month follow-up; *†, with respect to baseline and 3-month follow-up (post hoc analysis using Tukey’s test).

SBP, systolic blood pressure; DBP, diastolic blood pressure; Wt, body weight, FBS, fasting blood sugar; HDL. High-density lipoprotein cholesterol; ACR, albumin-creatinine ratio

### Affect of early control of metabolic syndrome on the albumin:creatinine ratio

**Fig 1:**
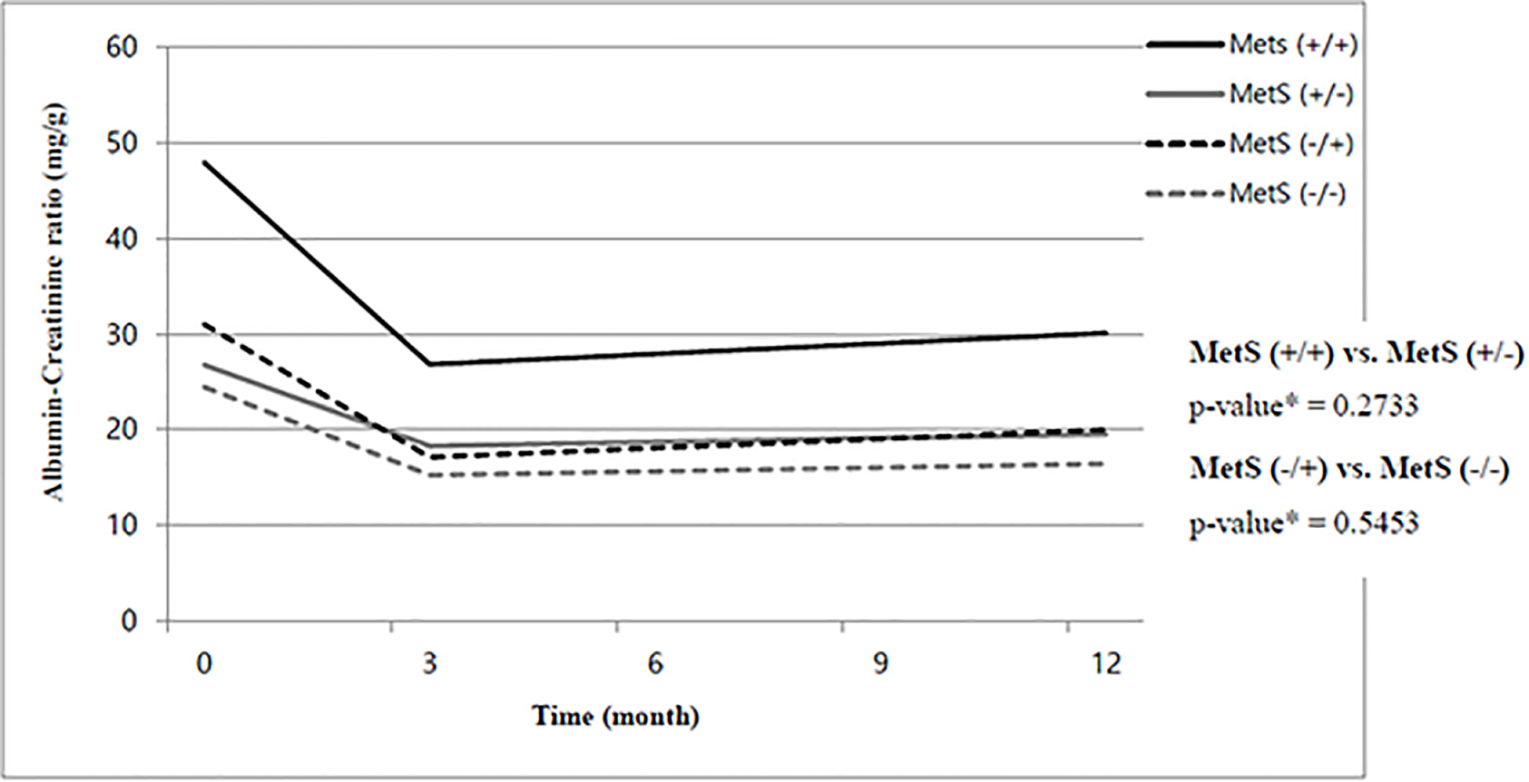
Change in the ACR (baseline, 3-months, 1-year follow-up) among the four study groups. **The ACR universally decreased in all patients irrespective with the MetS**. * p-value: < 0.05 using repeated measure ANOVA with Bonferroni correction of overall ACRs (baseline, 3-months, 1-year follow-up). ACR, albumin creatine ration; MetS, metabolic syndrome.

Although all four groups exhibited different changes in various metabolic risk factors, as described above, the ACR universally decreased in all patients (Fig 1), and the decrease was significant in both the MetS (+/+) and MetS (-/-) groups. Additionally, the prevalence of microalbuminuria decreased at 3 months and remained decreased for 1 year in all groups except the MetS (-/+) group (Table 3). Comparing the ACR in the MetS (+/+) and MetS (+/-) groups at 1 year, the ACR was significantly lower in the MetS (+/-) group (30.1 ± 123.8 vs. 19.5 ± 49.3, respectively, p = 0.0101, independent t-test with Bonferroni correction); metabolic syndrome was also corrected at 3 months in this group but not in the MetS (+/+) group. The difference between these groups, however, was no longer significant after adjusting for age, sex, body mass index, diabetes mellitus, SBP at 3 month, and baseline ACR (S1 Fig) in ANCOVA model. There was also no significant difference in the ACR at 1 year between the MetS (-/+), and MetS (-/-) groups (20.0 ± 62.0 vs 16.4 ± 67.6, respectively; p = 0.3765, independent t-test with Bonferroni correction) even though the MetS (-/+) group was characterized by newly developed MetS.

**Fig 2:**
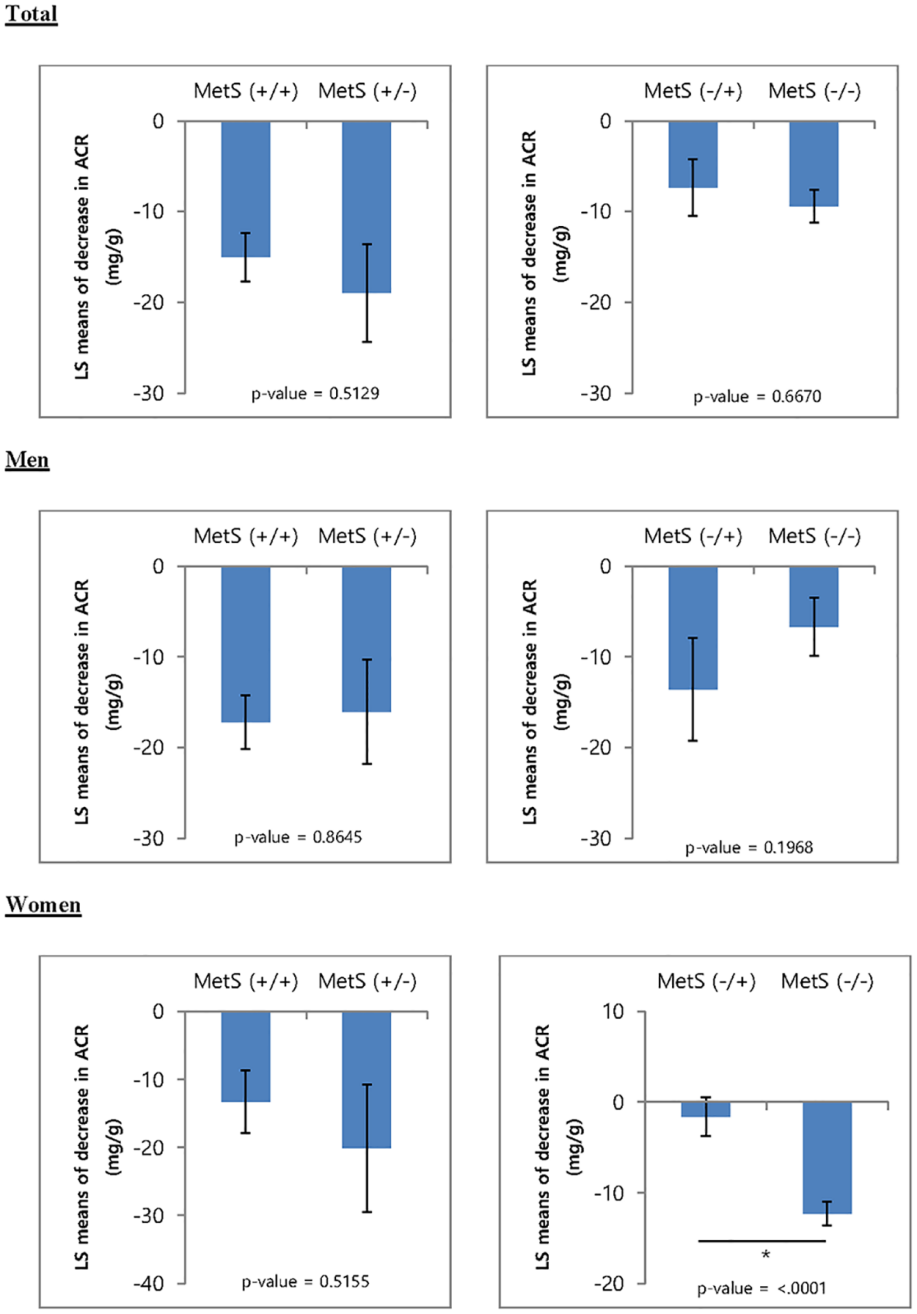
Comparison of the decrease in the ACR during 1 year between groups. The ACR of the MetS (-/-) group was significantly greater than that of the MetS (-/+) group in women (p < 0.0001), but not in men. Age, sex, body mass index, diabetes mellitus, ΔSBP (Base-1 year), and baseline ACR were adjusted and calculated using the least square means (LS means). p-value, comparison of LS means of decrease in ACR between group using ANCOVA model.

Because blood pressure changes during study period may directly influence the ACR, we used the adjusted LS means to compare the decrease in the ACR between groups using ANCOVA model after adjusting for age, sex, body mass index, diabetes mellitus, ΔSBP (baseline SBP– 1-year SBP), and baseline ACR and then compared the ΔACR between the MetS (+/+) and MetS (+/-) groups and between the MetS (-/+) and MetS (-/-) groups. Although there was no significant difference in the ΔACR between the four groups, the decrease in the ACR was greater in the MetS (+/-) group than in the MetS (+/+) group; likewise, the decrease in the ACR was greater in the MetS (-/-) group that in the MetS (-/+) group. These differences were more prominent in women than in men, and the decrease in ACR of the MetS (-/-) group was significantly greater than that of the MetS (-/+) group in women (-12.3 ± 1.3 vs. -1.64 ± 2.1, p < 0.0001, LSmeans in ANCOVA), but not in men (-6.7 ± 3.2 vs. -13.6 ± 5.7, p = 0.1968, LSmeans in ANCOVA; see Fig 2). In addition, among women, more MetS(-/+) patients experienced microalbuminuria than MetS(-/-) at 1 year (odds ratio 3.40; 95% confidence interval 1.60–7.17), but not in men (odds ratio 1.11; 95% confidence interval 0.60–2.06) (S2 Table). When grouping persistent MetS (+/+) and non-MetS (+/+), the baseline, 3 month, and 1 year ACR was persistently high in the MetS (+/+) group (Table 4).

**Table 4:** Comparison of the ACR between the MetS (+/+) and non-MetS (+/+) groups.

	MetS (+/+) (n = 1,460)	non-MetS (+/+) (n = 1,790)	p-value
Baseline	47.94 ± 192.72	26.28 ± 94.76	<.0001
3-month	26.84 ± 96.29	16.27 ± 50.76	0.0002
1-year	30.11 ± 123.76	17.81 ± 62.95	0.0006

mean ± SD; ACR, albumin:creatinine ratio; MetS, metabolic syndrome

p-value, Independent t-test for comparing two group.

## Discussion

Patients undergoing treatment with fimasartan for 1 year had a significant reduction in urinary albumin excretion in conjunction with a marked drop in blood pressure, despite no changes in the prevalence of MetS. Interestingly, in women, but not in men, increased metabolic risk in the first 3 months influenced microalbuminuria at 1 year.

### The effect of fimasartan on the incidence of metabolic syndrome

According to the Korean National Health and Nutrition Examination Survey, the prevalence of metabolic syndrome is increasing[23]. Similarly, the ARIRANG study showed that over a duration of about 2.6 years, 18.4% of men and 16.4% of women naturally developed metabolic syndrome. The ARIRANG study also showed that patients who developed metabolic syndrome experienced an increase in BMI, waist circumference, blood pressure, and fasting glucose and a decrease in HDL cholesterol, consistent with the overall trend of the general Korean population due to urbanization[24]. This indicates that the increased prevalence of the metabolic syndrome is not due to an increased incidence of hypertension but rather due to changes in lifestyle.

Choosing the appropriate treatment regimen for blood pressure control is important. Due to secondary and/or pleiotropic effects, CCBs and ARBs are metabolically neutral and usually chosen to treat high blood pressure[25]. Our study participants experienced a reduction in factors that are largely determined by lifestyle changes, such as high triglycerides, low HDL, and high fasting glucose for the first 3 months of treatment with the ARB fimasartan, but these changes did not persist for the entire yearlong study period. These factors are known to change in response to lifestyle modification even before physical changes, such as weight loss, are observed. Interestingly, despite the unchanged incidence of metabolic syndrome and the inconsistency in the worsening of metabolic factors over 1 year, the prevalence of microalbuminuria was clearly decreased and remained decreased for the entire year. This may be related to the renoprotective effects of ARBs that have been demonstrated in other studies[18, 26].

### Gender-associated differences in the effect of fimasartan treatment

In this study, changes in specific MetS risk factors and the degree of control of MetS differed between men and women. The effect of correcting MetS risk factors on microalbuminuria was also different between men and women. Similarly, the renin-angiotensin system (RAS), which when activated can contribute to the development of various kidney diseases and CVD irrespective of high blood pressure, is also influenced by sex. Estrogen suppresses the RAS by reducing blood pressure, angiotensin II levels, renin levels, and angiotensin-converting enzyme (ACE) activity, thus leading to vasodilation and vasorelaxation[27], while androgen has been reported to increase ACE activity and renin activity[28, 29]. Such differences in sex hormones between men and women may account for the efficacy of a given medication. A previous study demonstrated that a combination of estrogen and an ARB resulted in a greater reduction of proteinuria in a rat model of myocardial infarction[30]. Although many studies on sex hormones and RAS have been performed in animals under ideal conditions, and thus may not accurately reflect the clinical setting, we nevertheless hypothesize that diseases related to RAS would be greatly influenced by gender via the above-described mechanisms. However, little is known about how lifestyle modification is influenced by hormonal differences in treating vascular diseases. Therefore, additional research is needed to determine appropriate gender-specific treatment protocols for metabolic syndrome and related diseases.

### Limitations

This study has several limitations. First, this is an observational study, and although the participants were not given any additional instructions other than medication use, participants may have modified other lifestyle factors by their own choice at any point during the study. Thus, participants’ and physicians' individual actions, which were mostly not controlled in this study, might reflect more a daily basis. Second, there was no control group in this study, and patients were not randomized; therefore, it is impossible to compare the effects of fimasartan on metabolic syndrome and microalbuminuria to those of antihypertensive medications of other categories or to no treatment at all. Finally, most of the study participants had mild hypertension without overt cardiovascular diseases; therefore, only a small number of patients had microalbuminuria at baseline and the change in the ACR during the study period was very subtle. However, despite low ACRs, we observed significant differences in the ACR across the four study groups, suggesting that metabolic syndrome does indeed affect the incidence of microalbuminuria.

## Conclusion

The five components of metabolic syndrome are conveniently used for risk stratification in clinic, but even the prevalence of different combinations of these factors seems to vary by gender and age. Patients who were treated with fimasartan for 1 year showed a significant reduction in blood pressure and urinary albumin excretion, demonstrating that control of metabolic syndrome through ARB-effected blood pressure reduction is an effective means to prevent organ damage. Although blood pressure, other MetS risk factors, and microalbuminuria improved with fimasartan treatment in all four groups, the change in triglycerides and HDL was signficant only in women. Therefore, in addition to consistent control of blood pressure, we recommend lifestyle changes directed towards improving these two factors for the prevention of organ damage and future CVDs in women diagnosed with metabolic syndrome.

## Supporting information

S1 Fig Comparison of the 1-year ACR between the MetS (+/+) and MetS (+/-) groups and between the MetS (-/+) and MetS (-/-) groups. Age, Sex, Body mass index, Diabetes mellitus, 3 month SBP, Baseline ACR were adjusted.
p-value, comparsion fo LS means of ACR between group using ANCOVA model.
(TIF)

S1 Table Frequency of antihypertensive and other drugs taken with fiamsartan at baseline, 3-month and 1-year. p-value; changes in metabolic components were assessed with repeated measures ANOVA.
Comparisons significant at the α = 0.05 level are indicated by *, difference with respect to baseline; †, with respect to 3-month follow-up; *†, with respect to baseline and 3-month follow-up. HDL-C, High density lipoprotein cholesterol; ACE inhibitor, Angiotensin-converting enzyme inhibitor.
(DOCX)

S2 Table Comparison of the prevalence of microalbuminuria (ACR≥30 mg/g) after 1 Year between the MetS (+/+) group and the MetS (+/-), MetS (-/+) and MetS (-/-) groups. OR, Odds ratio; CI, confidence interval.
p-value, <0.05 indicates that the risk of microalbuminuria is significantly associated between group.
Logistic regression after adjusting covariates with age, diabetes mellitus, 1 year SBP and baseline ACR.
* OR represents adjusted odds ratio for the presence of microalbuminuria (ACR≥30 mg/g) after 1-year and the presence of MetS at 3-month follow-up after adjusting for the above covariates.
(DOCX)
