# Supplementary figures and images for: Gender-specific differences in the incidence of microalbuminuria in metabolic syndrome patients after treatment with fimasartan: The K-MetS study
Source: PLoS One. 2017 Dec 19;12(12):e0189342. doi: 10.1371/journal.pone.0189342 (PMC5736217; doi:10.1371/journal.pone.0189342)

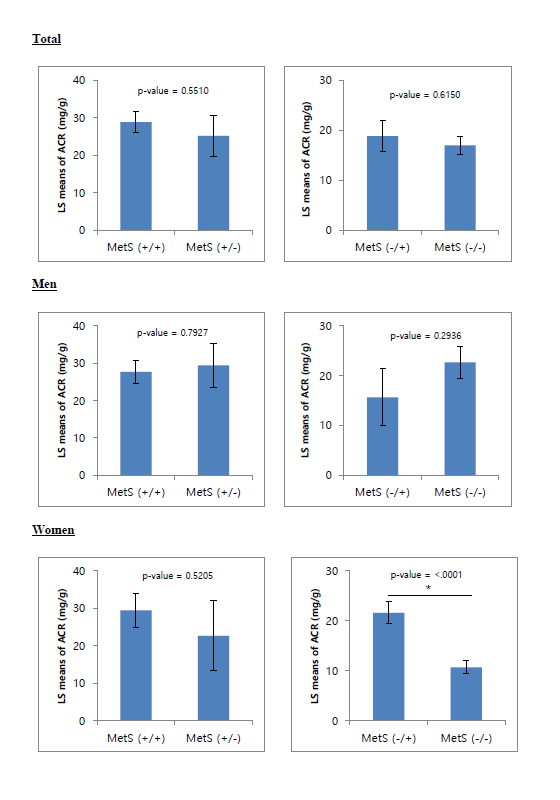

Supplement: S1 Fig — Age, Sex, Body mass index, Diabetes mellitus, 3 month SBP, Baseline ACR were adjusted. p-value, comparsion fo LS means of ACR between group using ANCOVA model. (TIF) [file pone.0189342.s001.tif]
