# Supplementary material for: Gender-specific differences in the incidence of microalbuminuria in metabolic syndrome patients after treatment with fimasartan: The K-MetS study
Source: PLoS One. 2017 Dec 19;12(12):e0189342. doi: 10.1371/journal.pone.0189342 (PMC5736217; doi:10.1371/journal.pone.0189342)
Supplement: S1 Table — p-value; changes in metabolic components were assessed with repeated measures ANOVA. Comparisons significant at the α = 0.05 level are indicated by *, difference with respect to baseline; †, with respect to 3-month follow-up; *†, with respect to baseline and 3-month follow-up. HDL-C, High density lipoprotein cholesterol; ACE inhibitor, Angiotensin-converting enzyme inhibitor. (DOCX) [file pone.0189342.s002.docx]

S1 table. Frequency of antihypertensive and other drugs taken with fiamsartan at baseline, 3-month and 1-year.

|  | Patients (N=3,250) | | | | Men (N=1,697) | | | | Women (N=1,553) | | | |
| --- | --- | --- | --- | --- | --- | --- | --- | --- | --- | --- | --- | --- |
|  | Baseline | 3-month | 1-year | P-value | Baseline | 3-month | 1-year | P-value | Baseline | 3-month | 1-year | P-value |
| Angiotensin receptor blocker | 733 (22.6) | - | - | - | 384 (11.8) | - | - | - | 349 (10.7) | - | - | - |
| ACE inhibitor | 57 (1.8) | 5 (0.2)* | 11 (0.3)* | <.0001 | 35 (1.1) | 2 (0.1)* | 6 (0.2)* | <.0001 | 22 (0.7) | 3 (0.1)* | 5 (0.2)* | <.0001 |
| β-blocker | 316 (9.7) | 201 (6.2)* | 204 (6.3)* | <.0001 | 179 (5.5) | 119 (3.7)* | 121 (3.7)* | <.0001 | 137 (4.2) | 82 (2.5)* | 83 (2.6)* | <.0001 |
| Calcium channel blocker | 979 (30.1) | 723 (22.2)* | 794 (24.4)*† | <.0001 | 542 (16.7) | 424 (13)* | 461 (14.2)*† | <.0001 | 437 (13.4) | 299 (9.2)* | 333 (10.2)*† | <.0001 |
| Diuretics | 149 (4.6) | 216 (6.6)* | 237 (7.3)* | <.0001 | 76 (2.3) | 106 (3.3)* | 112 (3.4)* | 0.0043 | 73 (2.2) | 110 (3.4)* | 125 (3.8)* | <.0001 |
| α-blocker | 13 (0.4) | 10 (0.3) | 11 (0.3) | 0.7416 | 7 (0.2) | 6 (0.2) | 9 (0.3) | 0.5300 | 6 (0.2) | 4 (0.1) | 2 (0.1) | 0.3792 |
| Antiplatelet agent | 603 (18.6) | 598 (18.4) | 656 (20.2)*† | 0.0009 | 349 (10.7) | 350 (10.8) | 386 (11.9)*† | 0.0028 | 254 (7.8) | 248 (7.6) | 270 (8.3) | 0.1641 |
| Anti-ischemic durgs | 95 (2.9) | 82 (2.5) | 68 (2.1)* | 0.0069 | 50 (1.5) | 42 (1.3) | 44 (1.4) | 0.3879 | 45 (1.4) | 40 (1.2) | 24 (0.7)*† | 0.0004 |
| Antidiabetics | 487 (15.0) | 488 (15.0) | 498 (15.3) | 0.6260 | 297 (9.1) | 295 (9.1) | 301 (9.3) | 0.8002 | 190 (5.8) | 193 (5.9) | 197 (6.1) | 0.7430 |
| Insulin | 34 (1.0) | 29 (0.9) | 28 (0.9) | 0.3491 | 15 (0.5) | 14 (0.4) | 13 (0.4) | 0.7933 | 19 (0.6) | 15 (0.5) | 15 (0.5) | 0.2614 |
| Drug for reduced HDL-C | 747 (23.0) | 869 (26.7)* | 1037 (31.9)*† | <.0001 | 365 (11.2) | 413 (12.7)* | 503 (15.5)*† | <.0001 | 382 (11.8) | 456 (14.0)* | 534 (16.4)*† | <.0001 |
| Drug for elevated triglyceride | 99 (3.0) | 114 (3.5) | 123 (3.8) | 0.0876 | 68 (2.1) | 85 (2.6) | 86 (2.6) | 0.1002 | 31 (1) | 29 (0.9) | 37 (1.1) | 0.2558 |

p-value; changes in metabolic components were assessed with repeated measures ANOVA.

Comparisons significant at the α = 0.05 level are indicated by *, difference with respect to baseline; †, with respect to 3-month follow-up; *†, with respect to baseline and 3-month follow-up. HDL-C, High density lipoprotein cholesterol; ACE inhibitor, Angiotensin-converting enzyme inhibitor.
