# Supplementary material for: Gender-specific differences in the incidence of microalbuminuria in metabolic syndrome patients after treatment with fimasartan: The K-MetS study
Source: PLoS One. 2017 Dec 19;12(12):e0189342. doi: 10.1371/journal.pone.0189342 (PMC5736217; doi:10.1371/journal.pone.0189342)
Supplement: S2 Table — OR, Odds ratio; CI, confidence interval. p-value, <0.05 indicates that the risk of microalbuminuria is significantly associated between group. Logistic regression after adjusting covariates with age, diabetes mellitus, 1 year SBP and baseline ACR. * OR represents adjusted odds ratio for the presence of microalbuminuria (ACR≥30 mg/g) after 1-year and the presence of MetS at 3-month follow-up after adjusting for the above covariates. (DOCX) [file pone.0189342.s003.docx]

S2 table. Comparison of the prevalence of microalbuminuria (ACR≥30 mg/g) after 1 Year between the MetS (+/+) group and the MetS (+/-), MetS (-/+) and MetS (-/-) groups.

| Group | Microalbuminuria (+) | Microalbuminuria (-) | | OR | | 95% CI | | p-value | Group | Microalbuminuria (+) | Microalbuminuria (-) | OR | 95% CI | p-value | |
| --- | --- | --- | --- | --- | --- | --- | --- | --- | --- | --- | --- | --- | --- | --- | --- |
| **Total** | | | | | | | | | | | | | | | |
| MetS (+) at baseline (n=1843, 56.7%) | | | | | | | | | MetS (-) at baseline (n=1407, 43.3%) | | | | | | |
| MetS (+/+) (n=1460) | 204 (14.0%) | 1256 (86.0%) | | 0.82 | | 0.56-1.22 | | 0.3433 | MetS (-/+)  (n=364) | 41 (11.3%) | 323 (88.7%) | 1.68 | 1.06-2.66 | 0.0260 | |
| MetS (+/-)  (n=383) | 44 (11.5%) | 339 (88.5%) | |  |  |  |  |  | MetS (-/-)  (n=1043) | 77 (7.4%) | 966 (92.6%) |  |  |  |  |
| **Men** | | | | | | | | | | | | | | | |
| MetS (+) at baseline (n=958, 56.5%) | | | | | | | | | MetS (-) at baseline (n=739, 43.5%) | | | | | | |
| MetS (+/+)  (n=750) | 102 (13.6%) | 648 (86.4%) | 0.75 | | | 0.45-1.24 | | 0.2677 | MetS (-/+)  (n=182) | 23 (12.6%) | 159 (87.4%) | 1.11 | 0.60-2.06 | 0.7387 | |
| MetS (+/-)  (n=208) | 26 (12.5%) | 182 (87.5%) |  |  |  |  |  |  | MetS (-/-)  (n=557) | 55 (9.9%) | 502 (90.1%) |  |  |  |  |
| **Women** | | | | | | | | | | | | | | | |
| MetS (+) at baseline (n=885, 57.0%) | | | | | | | | | MetS (-) at baseline (n=668, 43.0%) | | | | | |  |
| MetS (+/+)  (n=710) | 102 (13.4%) | 608 (85.6%) | | | 0.96 | | 0**.**51-1.81 | 0.9051 | MetS (-/+)  (n=182) | 18 (9.9%) | 164 (90.1%) | 3.40 | 1.61-7.17 | 0.0013 |  |
| MetS (+/-)  (n=175) | 18 (10.3%) | 157 (89.7%) | | |  |  |  |  | MetS (-/-)  (n=486) | 22 (4.5%) | 464 (95.5%) |  |  |  |  |

OR, Odds ratio; CI, confidence interval.

p-value, <0.05 indicates that the risk of microalbuminuria is significantly associated between group.

Logistic regression after adjusting covariates with age, diabetes mellitus, 1 year SBP and baseline ACR.

* OR represents adjusted odds ratio for the presence of microalbuminuria (ACR≥30 mg/g) after 1-year and the presence of MetS at 3-month follow-up after adjusting for the above covariates
